# Adalimumab exhibits superiority over etanercept in terms of a numerically higher response rate and equivalent adverse events: A real‐world finding

**DOI:** 10.1002/iid3.1166

**Published:** 2024-02-01

**Authors:** Zhe Yu, Ling Gao, Yinshan Zang, Lu Cheng, Wenjia Gao, Yan Xu

**Affiliations:** ^1^ Department of Rheumatology & Immunology The Affiliated Suqian First People's Hospital of Nanjing Medical University Suqian China

**Keywords:** adalimumab, efficacy, etanercept, rheumatoid arthritis, safety

## Abstract

**Introduction:**

Adalimumab (ADA) and etanercept (ETN) are the most commonly applied biologics for rheumatoid arthritis (RA) management in China; however, the evidence regarding their superiority is controversial. In addition, in real‐world clinical settings, many factors may affect the application of these agents, such as dosage and administration period. Therefore, the present real‐world study aimed to compare the efficacy and safety of ADA and ETN treatment in RA patients via the propensity score matching method.

**Methods:**

In total, 105 RA patients receiving ADA (*n* = 66) or ETN (*n* = 39) were reviewed in this retrospective study. The propensity score matching method was used to eliminate discrepancies in baseline features. Clinical response, low disease activity (LDA), and remission were evaluated based on the DAS28.

**Results:**

Before propensity score matching, compared with ETN, ADA yielded higher rates of clinical response at W24 (97.0% vs. 84.6%, *p* = .021), LDA at W12 (78.8% vs. 51.3%, *p* = .003), and remission at W24 (75.8% vs. 46.2%, *p* = .002). After propensity score matching, compared with ETN, ADA only achieved a higher rate of clinical response at W24 (96.3% vs. 77.8%, *p* = .043), whereas the rates of LDA and remission were not different between ADA and ETN treatments at any time point (all *p* > .05). In addition, the incidence of adverse events was not significantly different between the ADA and ETN treatments (all *p* > .05).

**Conclusion:**

ADA shows superiority over ETN in terms of a numerically greater response rate and equivalent adverse events.

## Graphical abstract

Before propensity‐score matching, adalimumab achieves higher rates of clinical response, low disease activity, and remission than etanercept in rheumatoid arthritis patients. After matching, adalimumab still realizes a higher rate of clinical response than etanercept to some extent with equivalent safety in rheumatoid arthritis patients

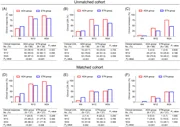

## 1 INTRODUCTION

Rheumatoid arthritis (RA) is an autoimmune disease that manifests as symmetric polyarthritis and may result in damage to joints and periarticular structures.^1^, ^2^, ^3^ Generally, RA is more common in females than in males, with a general prevalence of 0.2% in China.^4^, ^5^, ^6^ According to the 2021 American College of Rheumatology guidelines, conventional synthetic disease‐modifying antirheumatic drugs (csDMARDs) (methotrexate) with or without glucocorticoids are the first‐line therapy for the treatment of RA.^7^ Unfortunately, some RA patients fail first‐line treatment and may even harbor risk factors for poor prognosis, such as the presence of autoantibodies, high disease activity, and early joint damage on radiography.^7^, ^8^, ^9^, ^10^ In such cases, biological DMARDs (bDMARDs), which are advantageous because of their rapid onset of action and inhibition of radiographic progression, should be applied.^11^, ^12^, ^13^ Tumor necrosis factor (TNF) inhibitors are bDMARDs that have satisfactory efficacy in treating RA patients.^14^, ^15^, ^16^ However, slight differences exist among the TNF inhibitors; therefore, comparisons of their efficacy are needed to determine the ideal regimen for the treatment of RA.

Adalimumab (ADA), a fully humanized monoclonal antibody, and etanercept (ETN), a fusion protein, both of which are two common TNF inhibitors.^17^, ^18^, ^19^ Notably, ADA binds to both transmembrane and soluble TNF‐α and has a long duration of action, whereas ETN barely binds to transmembrane TNF‐α.^20^ Several clinical studies have explored the efficacy of ADA and ETN in RA patients, and they indicate that both ADA and ETN are effective in these patients.^21^, ^22^, ^23^ Apart from that, a few studies have indirectly compared the efficacy of ADA and ETN in RA patients, and these studies have shown that the efficacy of ADA and ETN might not differ for the treatment of RA.^24^, ^25^, ^26^ However, evidence regarding the head‐to‐head comprehensive comparison of efficacy between ADA and ETN in RA patients in a real‐world clinical setting is still lacking.

Accordingly, the present real‐world study was conducted with the aim of directly comparing the efficacy and safety of ADA versus ETN for the treatment of RA.

## 2 MATERIALS AND METHODS

### 2.1 Patients

Between June 2015 and December 2022, 105 RA patients who received ADA or ETN treatment in our hospital were deemed eligible for this real‐world, retrospective study. The eligibility criteria for inclusion were as follows: (a) diagnosed with RA; (b) had an active disease status according to the Disease Activity Score‐28 with an erythrocyte sedimentation rate (DAS28_ESR_) > 3.2^27^; (c) were older than 18 years; (d) had received ADA or ETN treatment for 6 months; and (e) had accessible and available data for study analysis. The exclusion criteria were as follows: (a) had acute or chronic infections or a previous history of active tuberculosis; (b) had malignancies; (c) were positive for hepatitis B antigen or hepatitis C antibody; (d) had participated in other clinical studies within 3 months before administration; and (e) were females who were pregnant or lactating. The study was approved by the Ethics Committee of Suqian First People's Hospital (approval number: 20230005). The eligible patients signed informed consent forms.

### 2.2 Data collection

Demographics, disease history, disease duration, disease status, disease activity indices, and treatment information of the RA patients were obtained. The patients received ADA or ETN treatment for 6 months. The ADA contained Humira (Abbvie, Inc.), Anjianning (Hisun Biopharmaceutical Co., Ltd), and Sulixin (Innovent Biopharmaceutical Co., Ltd). The ETN contained Enbrel (Pfizer, Inc.), Qiangke (Celgen Biopharmaceutical Co., Ltd), and Yisaipu (Guojian Pharmaceutical Co., Ltd). In addition, data on the combination of csDMARDs were also collected. The patients who received ADA treatment were considered the ADA group, while the patients who received ETN treatment were considered the ETN group. For efficacy assessment, the DAS28_ESR_ was scored before treatment (W0) and at 4 weeks (W4), 12 weeks (W12), and 24 weeks (W24) after treatment. Afterward, clinical response (DAS28_ESR_ improvement ≥ 1.2), clinical low disease activity (LDA) (DAS28_ESR_ ≤ 3.2), and clinical remission (DAS28_ESR_ ≤ 2.6) were evaluated.^11^, ^27^, ^28^ Additionally, adverse events during treatment were recorded for safety assessment.

### 2.3 Statistics

Propensity score matching analysis was used to match the patients in the ADA group and the patients in the ETN group with a 1:1 ratio. The closest match method was adopted, and age, disease duration, DAS28_ESR_ score, and clinical disease activity index (CDAI) score were severed as covariates. The caliper value was set as 0.02. SPSS v22.0 (IBM Crop.) was used for analysis. GraphPad Prism v7.0 (GraphPad Software, Inc.) was used for graphing. Regarding sample size determination, as this was a real‐world study, we did not calculate the sample size but included as many patients as possible. Normality was analyzed using the Kolmogorov‒Smirnov test. Comparisons between groups were conducted using the *t* test, Wilcoxon rank‐sum test, *χ* ^2^ test, or Fisher's exact test. Comparisons over time were analyzed using the Friedman test or *χ* ^2^ test for trend. *p* < .05 indicated statistical significance.

## 3 RESULTS

### 3.1 Clinical features in the ADA group and the ETN group

In the unmatched cohort, there were 66 RA patients in the ADA group and 39 RA patients in the ETN group. The mean ages of the ADA group and ETN group were 50.7 ± 12.2 and 56.9 ± 12.0 years, respectively (*p* = .013). There were 18 (27.3%) males and 48 (72.7%) females in the ADA group and nine (23.1%) males and 30 (76.9%) females in the ETN group (*p* = .635). Notably, the disease duration was longer in the ETN group than in the ADA group (*p* < .001). In addition, the proportions of patients who were rheumatoid factor‐positive (*p* = .049) and anticitrullinated protein autoantibody‐positive (*p* = .049) were greater in the ADA group than in the ETN group. Furthermore, the median (interquartile range [IQR]) tender joint count (*p* = .008), swollen joint count, erythrocyte sedimentation rate (ESR) (*p* = .002), C‐reactive protein (*p* = .001), DAS28_ESR_ (*p* = .001), patient's global assessment score (*p* = .001), physician's global assessment score (*p* = .001), and CDAI score (*p* = .001) were lower in the ADA group than in the ETN group. All RA patients received the combination of csDMARDs and the proportion of RA patients who received different types of csDMARDs combinations was not different between the two groups (*p* = .056). In detail, in the ADA group, 59 (89.4%), five (7.6%), and two (3.0%) patients received methotrexate, leflunomide, and others, respectively, whereas no patients received hydroxychloroquine; in the ETN group, 29 (74.4%), three (7.7%), 2 (5.1%), and five (12.8%) patients received methotrexate, leflunomide, hydroxychloroquine, and others, respectively (Table 1).

**Table 1:** Clinical characteristics of the RA patients.

Items	Unmatched cohort				Matched cohort			
	RA patients (*N* = 105)	ADA group (*N* = 66)	ETN group (*N* = 39)	*p*	RA patients (*N* = 54)	ADA group (*N* = 27)	ETN group (*N* = 27)	*p*
Age (years), mean ± SD	53.0 ± 12.5	50.7 ± 12.2	56.9 ± 12.0	.013	55.4 ± 11.7	55.3 ± 11.1	55.4 ± 12.4	.972
Gender, No. (%)				.635				.154
Male	27 (25.7)	18 (27.3)	9 (23.1)		19 (35.2)	12 (44.4)	7 (25.9)	
Female	78 (74.3)	48 (72.7)	30 (76.9)		35 (64.8)	15 (55.6)	20 (74.1)	
BMI (kg/m^2^), median (IQR)	22.6 (20.6–25.6)	22.6 (20.5–24.5)	23.5 (21.1–28.3)	.168	23.5 (21.2–26.9)	24.3 (21.5–26.5)	23.2 (20.8–27.1)	.545
History of NSAID, No. (%)				1.000				1.000
No	2 (1.9)	1 (1.5)	1 (2.6)		1 (1.9)	0 (0.0)	1 (3.7)	
Yes	103 (98.1)	65 (98.5)	38 (97.4)		53 (98.1)	27 (100.0)	26 (96.3)	
History of GC, No. (%)				1.000				1.000
No	3 (2.9)	2 (3.0)	1 (2.6)		1 (1.9)	0 (0.0)	1 (3.7)	
Yes	102 (97.1)	64 (97.0)	38 (97.4)		53 (98.1)	27 (100.0)	26 (96.3)	
History of csDMARDs, No. (%)				1.000				.491
No	6 (5.7)	4 (6.1)	2 (5.1)		2 (3.7)	0 (0.0)	2 (7.4)	
Yes	99 (94.3)	62 (93.9)	37 (94.9)		52 (96.3)	27 (100.0)	25 (92.6)	
History of tsDMARDs, No. (%)				.363				.142
No	92 (87.6)	56 (84.8)	36 (92.3)		45 (83.3)	20 (74.1)	25 (92.6)	
Yes	13 (12.4)	10 (15.2)	3 (7.7)		9 (16.7)	7 (25.9)	2 (7.4)	
History of bDMARDs, No. (%)				.143				1.000
No	101 (96.2)	65 (98.5)	36 (92.3)		53 (98.1)	26 (96.3)	27 (100.0)	
Yes	4 (3.8)	1 (1.5)	3 (7.7)		1 (1.9)	1 (3.7)	0 (0.0)	
Disease duration (years), median (IQR)	3.5 (1.7–15.0)	2.6 (1.0–5.4)	10.7 (4.0–22.0)	<.001	9.2 (3.2–23.3)	10.0 (3.2–24.5)	8.4 (3.1–15.0)	.556
RF, No. (%)				.049				.236
Negative	3 (2.9)	0 (0.0)	3 (7.7)		3 (5.6)	0 (0.0)	3 (11.1)	
Positive	102 (97.1)	66 (100.0)	36 (92.3)		51 (94.4)	27 (100.0)	24 (88.9)	
ACPA, No. (%)				.049				.236
Negative	3 (2.9)	0 (0.0)	3 (7.7)		3 (5.6)	0 (0.0)	3 (11.1)	
Positive	102 (97.1)	66 (100.0)	36 (92.3)		51 (94.4)	27 (100.0)	24 (88.9)	
Tender joint count, median (IQR)	6.0 (5.0–11.5)	6.0 (5.0–8.0)	10.0 (5.0–15.0)	.008	8.5 (5.0–15.0)	8.0 (6.0–16.0)	9.0 (5.0–15.0)	.514
Swollen joint count, median (IQR)	4.0 (2.0–7.0)	3.0 (2.0–5.0)	5.0 (3.0–10.0)	.002	5.0 (2.8–9.0)	5.0 (3.0–10.0)	5.0 (2.0–8.0)	.682
ESR (mm/h), median (IQR)	65.0 (45.9–91.8)	52.2 (45.2–79.3)	88.3 (58.4–98.4)	.001	86.5 (58.6–99.7)	84.2 (58.6–104.0)	88.3 (58.4–96.5)	.890
CRP (mg/L), median (IQR)	36.3 (24.0–63.6)	26.3 (22.8–53.5)	52.5 (26.5–76.9)	.001	49.5 (28.8–76.2)	48.6 (29.6–76.3)	49.7 (25.7–76.0)	.924
DAS28_ESR_, median (IQR)	5.3 (4.9–6.4)	5.1 (4.8–5.8)	6.2 (5.2–7.0)	.001	6.1 (5.1–6.7)	6.0 (5.3–7.0)	6.1 (5.1–6.6)	.723
PGA score, median (IQR)	6.0 (5.0–7.0)	5.0 (4.0–7.0)	7.0 (5.0–8.0)	.001	6.5 (5.0–7.0)	7.0 (5.0–7.0)	6.0 (5.0–8.0)	.979
PhGA score, median (IQR)	5.0 (4.0–7.0)	5.0 (4.0–6.3)	6.0 (5.0–7.0)	.001	6.0 (5.0–7.0)	6.0 (5.0–7.0)	6.0 (5.0–7.0)	.986
CDAI, median (IQR)	21.0 (16.0–33.0)	18.0 (15.0–25.3)	31.0 (18.0–39.0)	.001	29.0 (18.0–35.3)	29.0 (19.0–36.0)	29.0 (17.0–34.0)	.782
Combination of csDMARDs, No. (%)	105 (100.0)	66 (100.0)	39 (100.0)	–		27 (100.0)	27 (100.0)	–
Type of csDMARDs combination, No. (%)				.056				.274
MTX	88 (83.8)	59 (89.4)	29 (74.4)		45 (83.3)	25 (92.6)	20 (74.1)	
LEF	8 (7.6)	5 (7.6)	3 (7.7)		4 (7.4)	1 (3.7)	3 (11.1)	
HCQ	2 (1.9)	0 (0.0)	2 (5.1)		2 (3.7)	0 (0.0)	2 (7.4)	
Others	7 (6.7)	2 (3.0)	5 (12.8)		3 (5.6)	1 (3.7)	2 (7.4)	

Abbreviations: ACPA, anticitrullinated protein autoantibody; ADA, adalimumab; bDMARDs, biologic disease‐modifying antirheumatic drugs; BMI, body mass index; CDAI, clinical disease activity index; CRP, C‐reactive protein; csDMARDs, conventional synthetic disease‐modifying antirheumatic drugs; DAS28_ESR_, 28‐joint disease activity score using the ESR; ESR, erythrocyte sedimentation rate; ETN, etanercept; GC, glucocorticoid; HCQ, hydroxychloroquine; IQR, interquartile range; LEF, leflunomide; MTX, methotrexate; NSAID, nonsteroidal anti‐inflammatory drugs; PGA, patient's global assessment; PhGA, physician's global assessment; RA, rheumatoid arthritis; RF, rheumatoid factor; tsDMARDs, targeted synthetic disease‐modifying antirheumatic drugs.

**Figure 1:**
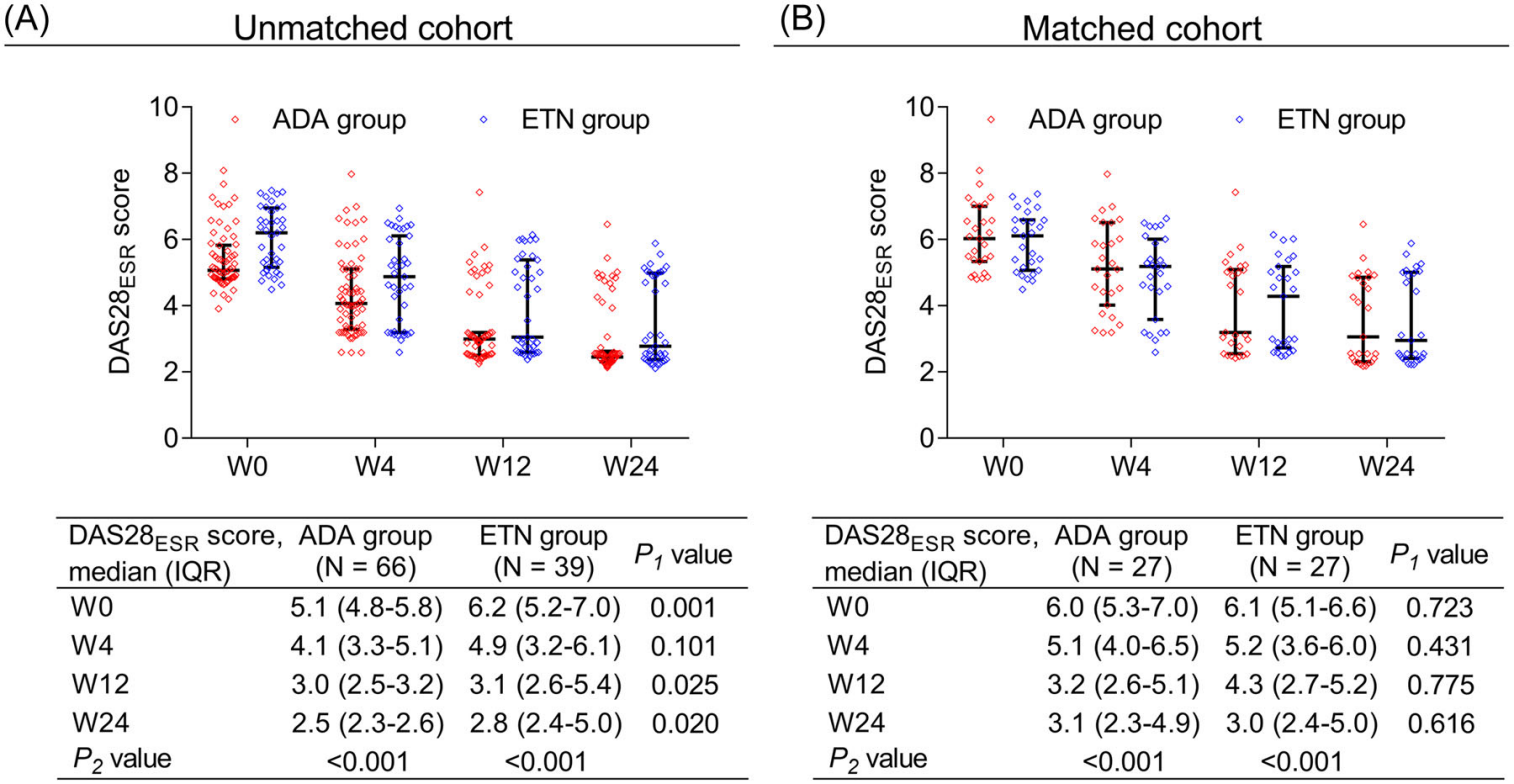
28‐joint disease activity score using the erythrocyte sedimentation rate (DAS28_ESR_) comparison between the adalimumab (ADA) group and etanercept (ETN) group. Comparison of the DAS28_ESR_ at W0, W4, W12, and W24 between the ADA group and the ETN group in the unmatched cohort (A) and matched cohort (B).

After adjustment by propensity score matching analysis, patients were matched at a 1:1 ratio, and 27 patients were in the ADA group, whereas 27 patients were in the ETN group. The matching procedure was exhibited in Supporting Information S1: Figure 1. The mean ages of the ADA group and ETN group were 55.3 ± 11.1 years and 55.4 ± 12.4 years, respectively (*p* = .972). There were 12 (44.4%) males and 15 (55.6%) females in the ADA group, and seven (25.9%) males and 20 (74.1%) females in the ETN group (*p* = .154). Notably, all clinical characteristics were balanced between the two groups after adjustment (all *p* > .05). The proportion of RA patients who received different types of csDMARDs combinations was not different between the two groups (*p* = .274). In detail, in the ADA group, 25 (92.6%), 1 (3.7%), and 1 (3.7%) patients received methotrexate, leflunomide, and others, respectively, but no patients received hydroxychloroquine; in the ETN group, 20 (74.1%), 3 (11.1%), 2 (7.4%), and 2 (7.4%) patients received methotrexate, leflunomide, hydroxychloroquine, and others, respectively. The detailed clinical information is listed in Table 1.

### 3.2 Comparison of the DAS28_ESR_ between the ADA group and the ETN group

In the unmatched group, the DAS28_ESR_ was decreased from W0 to W24 in both the ADA group (*p* < .001) and the ETN group (*p* < .001). In addition, the DAS28_ESR_ at W0 (median [IQR]: 5.1 [4.8–5.8] vs. 6.2 [5.2–7.0]) (*p* = .001), W12 (median [IQR]: 3.0 [2.5–3.2] vs. 3.1 [2.6–5.4]) (*p* = .025), and W24 (median [IQR]: 2.5 [2.3–2.6] vs. 2.8 [2.4–5.0]) (*p* = .020) were lower in the ADA group than in the ETN group. However, it remained unchanged at W4 between the two groups (median [IQR]: 4.1 [3.3–5.1] vs. 4.9 [3.2–6.1]) (*p* = .101) (Figure 1A).

**Figure 2:**
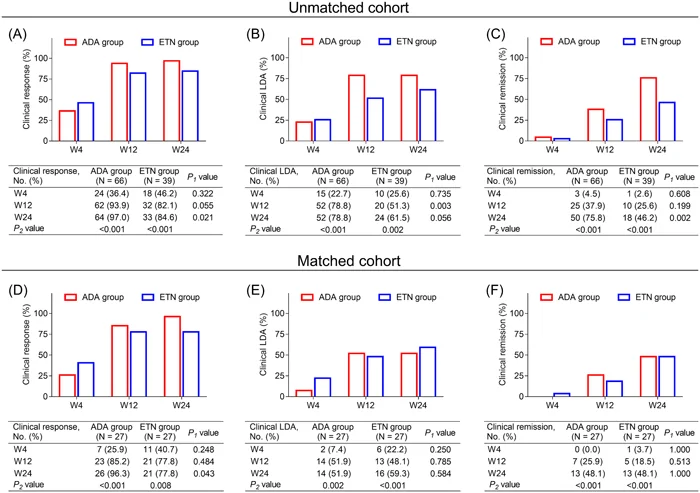
Clinical response, low disease activity (LDA), and remission rate comparisons between the adalimumab (ADA) group and the etanercept (ETN) group. Comparison of the clinical response (A), LDA (B), and remission (C) rates at W4, W12, and W24 between the ADA group and ETN group in the unmatched cohort; comparison of the clinical response (D), LDA (E), and remission (F) rates at W4, W12, and W24 between the ADA group and ETN group in the matched cohort.

In the matched cohort, the DAS28_ESR_ also showed a decreasing trend from W0 to W24 in the ADA group (*p* < .001) and the ETN group (*p* < .001). However, the DAS28_ESR_ did not significantly differ between the two groups at W0 (median [IQR]: 6.0 [5.3–7.0] vs. 6.1 [5.1–6.6]) (*p* = .723), W4 (median [IQR]: 5.1 [4.0–6.5] vs. 5.2 [3.6–6.0]) (*p* = .431), W12 (median [IQR]: 3.2 (2.6–5.1) vs. 4.3 [2.7–5.2]) (*p* = .775), and W24 (median [IQR]: 3.1 [2.3–4.9] vs. 3.0 [2.4–5.0]) (*p* = .616) (Figure 1B). The photographs of two RA patients before and after ADA or ETN treatment were shown in Supporting Information S2: Figure 2.

### 3.3 Comparison of clinical response, LDA, and remission rates between the ADA group and the ETN group

In the unmatched cohort, the clinical response, LDA, and remission rates increased from W4 to W24 in the ADA group and ETN group (all *p* < .05) (Figure 2A–C). In addition, the clinical response rate at W24 was greater in the ADA group than in the ETN group (97.0% vs. 84.6%) (*p* = .021) (Figure 2A). Moreover, the clinical LDA rate at W12 was greater in the ADA group than in the ETN group (78.8% vs. 51.3%) (*p* = .003) (Figure 2B). Additionally, the clinical remission rate at W24 was greater in the ADA group than in the ETN group (75.8% vs. 46.2%) (*p* = .002) (Figure 2C).

In the matched cohort, the clinical response, LDA, and remission rates increased from W4 to W24 in the ADA group and ETN group (all *p* < .05) (Figure 2D–F). Moreover, the clinical response rate at W24 was also greater in the ADA group than in the ETN group (96.3% vs. 77.8%) (*p* = .043) (Figure 2D). However, the clinical LDA rates at W4 (*p* = .250), W12 (*p* = .785), and W24 (*p* = .584) did not significantly differ between the two groups (Figure 2E). Moreover, the clinical remission rates at W4 (*p* = 1.000), W12 (*p* = .513), and W24 (*p* = 1.000) did not differ between the two groups (Figure 2F).

### 3.4 Subgroup analysis based on different combinations of csDMARDs

Subgroup analysis was carried out for the unmatched cohort. Among the RA patients receiving the combination of methotrexate, the DAS28_ESR_ at W0 was lower in the ADA group than in the ETN group (*p* = .008), whereas the clinical response, LDA, and remission rates at W4, W12, and W24 remained unchanged between the ADA group and the ETN group (all *p* > .05). Among the RA patients receiving the combination of other csDMARDs, the DAS28_ESR_ at W0 (*p* = .019), W12 (*p* = .040), and W24 (*p* = .040) was lower in the ADA group than in the ETN group. Moreover, the clinical response (*p* = .044) and remission (*p* = .015) rates at W24 were greater in the ADA group than in the ETN group (Table 2).

**Table 2:** Subgroup analysis of the DAS28_ESR_, clinical response, clinical LDA, and clinical remission in patients treated with different combinations of csDMARDs.

Items	Unmatched cohort			Matched cohort		
	ADA group (*N* = 66)	ETN group (*N* = 39)	*p*	ADA group (*N* = 27)	ETN group (*N* = 27)	*p*
Combination of MTX						
DAS28_ESR_, median (IQR)						
W0	5.1 (4.8–5.9)	6.1 (5.0–7.0)	.008	6.0 (5.4–7.0)	5.6 (5.0–6.8)	.283
W4	4.1 (3.3–5.1)	4.6 (3.2–5.8)	.414	5.1 (4.2–6.5)	4.8 (3.2–5.4)	.115
W12	3.0 (2.5–3.2)	3.0 (2.6–5.1)	.136	3.2 (2.6–5.1)	3.0 (2.6–4.8)	.648
W24	2.4 (2.3–2.7)	2.6 (2.4–4.6)	.227	3.1 (2.4–4.9)	2.6 (2.4–4.1)	.472
Clinical response, No. (%)						
W4	23 (39.0)	14 (48.3)	.406	7 (28.0)	9 (45.0)	.236
W12	55 (93.2)	26 (89.7)	.680	21 (84.0)	18 (90.0)	.678
W24	57 (96.6)	28 (96.6)	1.000	24 (96.0)	19 (95.0)	1.000
Clinical LDA, No. (%)						
W4	14 (23.7)	9 (31.0)	.606	2 (8.0)	6 (30.0)	.113
W12	46 (78.0)	17 (58.6)	.079	13 (52.0)	12 (60.0)	.592
W24	46 (78.0)	21 (72.4)	.601	13 (52.0)	15 (75.0)	.114
Clinical remission, No. (%)						
W4	3 (5.1)	1 (3.4)	1.000	0 (0.0)	1 (5.0)	.444
W12	24 (40.7)	9 (31.0)	.484	7 (28.0)	5 (25.0)	.821
W24	44 (74.6)	16 (55.2)	.066	12 (48.0)	12 (60.0)	.423
Combination of other csDMARDs						
DAS28_ESR_, median (IQR)						
W0	4.8 (4.7–5.3)	6.4 (5.3–6.8)	.019	5.6 (4.8–NA)	6.6 (5.6–6.6)	.242
W4	4.0 (3.6–5.1)	5.6 (4.2–6.5)	.079	4.5 (3.6–NA)	5.9 (5.2–6.4)	.242
W12	2.9 (2.8–3.2)	5.0 (3.0–6.0)	.040	3.8 (2.8–NA)	5.0 (4.8–6.0)	.143
W24	2.5 (2.3–2.6)	5.0 (2.7–5.3)	.040	3.6 (2.3–NA)	5.2 (4.7–5.6)	.143
Clinical response, No. (%)						
W4	1 (14.3)	4 (40.0)	.338	0 (0.0)	2 (28.6)	1.000
W12	7 (100.0)	6 (60.0)	.103	2 (100.0)	3 (42.9)	.444
W24	7 (100.0)	5 (50.0)	.044	2 (100.0)	2 (28.6)	.167
Clinical LDA, No. (%)						
W4	1 (14.3)	1 (10.0)	1.000	0 (0.0)	0 (0.0)	NA
W12	6 (85.7)	3 (30.0)	.050	1 (50.0)	1 (14.3)	.417
W24	6 (85.7)	3 (30.0)	.050	1 (50.0)	1 (14.3)	.417
Clinical remission, No. (%)						
W4	0 (0.0)	0 (0.0)	NA	0 (0.0)	0 (0.0)	NA
W12	1 (14.3)	1 (10.0)	1.000	0 (0.0)	0 (0.0)	NA
W24	6 (85.7)	2 (20.0)	.015	1 (50.0)	1 (14.3)	.417

Abbreviations: ADA, adalimumab; csDMARDs, conventional synthetic disease‐modifying antirheumatic drugs; DAS28_ESR_, 28‐joint disease activity score using the erythrocyte sedimentation rate; ETN, etanercept; IQR, interquartile range; LDA, low disease activity; MTX, methotrexate; NA, not available.

Subgroup analysis was also conducted for the matched cohort. For both the RA patients receiving the combination of methotrexate and those receiving other csDMARDs, the DAS28_ESR_ at W0, W4, W12, and W24, as well as the clinical response, LDA, and remission rates at W4, W12, and W24, remained unchanged between the ADA group and the ETN group (all *p* > .05) (Table 2).

### 3.5 Comparison of adverse events between the ADA group and the ETN group

In the unmatched cohort, the incidence of adverse events, including pulmonary infection, skin infection, callosity, and dermatophytes, did not significantly differ between the two groups (all *p* > .05). The adverse events that occurred in the ADA group were skin infection (3.0%), pulmonary infection (1.5%), callosity (1.5%), and dermatophyte (1.5%), whereas the adverse events that occurred in the ETN group were pulmonary (10.3%) and callosity (7.7%) (Table 3).

**Table 3:** Adverse events.

Items	Unmatched cohort				Matched cohort			
	RA patients (*N* = 105)	ADA group (*N* = 66)	ETN group (*N* = 39)	*p*	RA patients (*N* = 54)	ADA group (*N* = 27)	ETN group (*N* = 27)	*p*
Total, No. (%)	12 (11.5)	5 (7.5)	7 (18.0)	.123	8 (14.8)	3 (11.1)	5 (18.5)	.444
Pulmonary infection, No. (%)	5 (4.8)	1 (1.5)	4 (10.3)	.062	3 (5.6)	1 (3.7)	2 (7.4)	.552
Skin infection, No. (%)	2 (1.9)	2 (3.0)	0 (0.0)	.529	1 (1.9)	1 (3.7)	0 (0.0)	1.000
Callosity, No. (%)	4 (3.8)	1 (1.5)	3 (7.7)	.143	4 (7.4)	1 (3.7)	3 (11.1)	.610
Dermatophyte, No. (%)	1 (1.0)	1 (1.5)	0 (0.0)	1.000	0 (0.0)	0 (0.0)	0 (0.0)	–

Abbreviations: ADA, adalimumab; ETN, etanercept; RA, rheumatoid arthritis.

In the matched cohort, the incidence of adverse events did not differ between the two groups (all *p* > .05). The adverse events in the ADA group were pulmonary infection (3.7%), skin infection (3.7%), and callosity (3.7%), whereas the adverse events that occurred in the ETN group were callosity (11.1%) and pulmonary infection (7.4%) (Table 3).

## 4 DISCUSSION

Interestingly, this study revealed real‐world prescribing habits of Chinese physicians for the treatment of RA. Specifically, ADA (vs. ETN) was more likely to be prescribed by physicians for the treatment of RA. The reason for this difference might be that although ETN was launched earlier than ADA in China, its prescription was limited by medical insurance, and most RA patients were not able to receive this treatment, leading to a longer disease duration and greater disease activity at baseline.^29^, ^30^ However, after ADA was placed in the market, medical insurance policy also started to improve; hence, many patients choose to use ADA after early diagnosis of RA, resulting in a shorter disease duration and lower disease activity at baseline.^31^ In addition, these findings may provide an objective picture of Chinese physicians' prescribing habits. However, to objectively compare the efficacy of ADA versus ETN in RA patients, matched baseline clinical features were needed. Therefore, propensity score matching was conducted to eliminate the baseline clinical characteristics of RA patients receiving ADA and those receiving ETN.

Considering that ADA and ETN have slightly different mechanisms of action (ADA binds to both transmembrane and soluble TNF‐α, whereas ETN barely binds to transmembrane TNF‐α), it is necessary to compare the efficacy of these two drugs. Moreover, several previous studies have compared the effect of ADA versus ETN in patients with autoimmune diseases.^32^, ^33^, ^34^, ^35^, ^36^ For example, the Psoriasis Area and Severity Index score at month 12 is lower in psoriasis patients receiving ADA than in those receiving ETN.^37^ The present study further directly compared the therapeutic efficacy between the RA patients receiving ADA and those receiving ETN. In the unmatched cohort, ADA exhibited better efficacy than ETN for the treatment of RA. A possible reason could be that patients receiving ADA tended to have clinical features of shorter disease duration and lower disease activity, which might have resulted in a reduced incidence of drug resistance;^38^, ^39^ thus, ADA had better treatment efficacy than ETN. After adjustment, ADA also exerted satisfactory treatment efficacy to some extent compared with that of ETN in RA patients. The potential explanation for this difference could be that ETN was rarely bound to transmembrane TNF‐α, while ADA was not only bound to soluble TNF‐α but also transmembrane TNF‐α, which might lead to a better effect on inhibiting the interaction between TNF‐α and its receptor, resulting in a better effect on reducing disease activity.^20^, ^40^, ^41^ Conclusively, in this study, we discovered that ADA achieved better efficacy compared to ETN in RA patients, in both unmatched cohort and matched cohort. Some external and internal reasons might explain our findings. Regarding external reasons: (1) RA patients receiving ADA tended to have mild disease conditions compared to those receiving ETN, which might lead to a smaller risk of developing drug resistance and treatment failure.^42^ (2) ADA required a less frequent subcutaneous administration compared to ETN due to its longer half‐time; therefore, medication adherence to ADA might be better than ETN in RA patients.^40^ Regarding internal reason, ADA was a fully‐humanized monoclonal antibody, and ETN was a fusion protein; of note, ADA could bind to both soluble and transmembrane TNF‐α, whereas ETN was barely bound to transmembrane TNF‐α, leading to a better effect of ADA on inhibiting TNF‐α.^20^, ^40^ Thus, ADA had a satisfactory efficacy to a certain extent compared to ETN in RA patients, even after adjustment.

The safety profile of ADA and ETN is similar in patients with autoimmune diseases (such as psoriasis and psoriatic arthritis).^34^, ^35^ In accordance with these previous studies, the present study revealed that the occurrence rate of adverse events was not different between RA patients receiving ADA and those receiving ETN. One reason for this difference might be that both ADA and ETN can suppress the immune system by inhibiting TNF‐α, which might influence susceptibility to infections and ultimately contribute to the fact that the incidence of adverse events has remained unchanged between ADA and ETN.^43^ Additionally, according to previous studies, the common adverse events induced by ADA or ETN are respiratory and skin infections in RA patients.^44^, ^45^, ^46^, ^47^ In line with these previous studies, this study revealed that the adverse events were pulmonary infection (3.7%), skin infection (3.7%), and callosity (3.7%) in patients receiving ADA, and they were callosity (11.1%) and pulmonary infection (7.4%) in those receiving ETN. Notably, the incidence of adverse events was relatively low in RA patients receiving ADA or ETN, and most adverse events were manageable. These findings indicated that ADA and ETN were safe for use in RA patients.

Notably, the strengths of this study should be highlighted as follows: (1) Even though previous studies have compared the efficacy of ADA versus ETN in patients with autoimmune diseases, their baseline clinical characteristics are not well balanced, which would affect the reliability of their findings.^32^, ^48^ Thus, to eliminate the differences in baseline clinical features, this study applied propensity score matching to further compare the effect of ADA versus ETN on reducing disease activity in RA patients. (2) This was a real‐world clinical study that reflects real‐world patient medication use and the difference in efficacy between ADA and ETN with a high degree of external realism, which might provide an angle for both physicians and RA patients in the selection of treatment strategies.

Several limitations should be mentioned: (1) the sample size of this study was not large enough, which might affect the statistical power; (2) propensity score matching analysis was applied to balance the baseline differences between the two groups; however, this was a retrospective study, and other potential factors might influence RA patients' choice of medications, which would further affect our results. Therefore, our findings still needed validation by further randomized‐controlled trials; (3) selection and information bias would exist in this retrospective study; (4) the follow‐up duration could be expanded to compare the long‐term efficacy of ADA and ETN in RA patients; (5) direct efficacy and safety comparisons among different small‐molecule Janus kinase inhibitors, such as tofacitinib, baricitinib, filgotinib, and upadacitinib, in RA patients in real‐world settings deserved to be further investigated.

In summary, ADA achieves better treatment efficacy than ETN, and they have comparable safety profiles in RA patients. However, limited by the sample size of this study, our findings may not be solid enough. Thus, more large‐scale studies are required to validate the findings of this study.

## AUTHOR CONTRIBUTIONS

Zhe Yu, Ling Gao, and Yan Xu contributed to the conception and design of the study. Yinshan Zang, Lu Cheng, and Wenjia Gao contributed to the data acquisition. Zhe Yu and Ling Gao performed the statistical analysis and drafted the manuscript. Yinshan Zang, Lu Cheng, Wenjia Gao, and Yan Xu revised the manuscript. All the authors contributed to the article and approved the submitted version.

## CONFLICT OF INTEREST STATEMENT

The authors declare no conflict of interest.

## ETHICS STATEMENT

The study was approved by the Ethics Committee. The eligible patients signed informed consent.

## Supporting information


Supporting Information

Supporting Information

## Data Availability

The data sets used and/or analyzed during the current study are available from the corresponding author on reasonable request.
