# Supplementary figures and images for: Adalimumab exhibits superiority over etanercept in terms of a numerically higher response rate and equivalent adverse events: A real‐world finding
Source: Immun Inflamm Dis. 2024 Feb 1;12(2):e1166. doi: 10.1002/iid3.1166 (PMC10832310; doi:10.1002/iid3.1166)

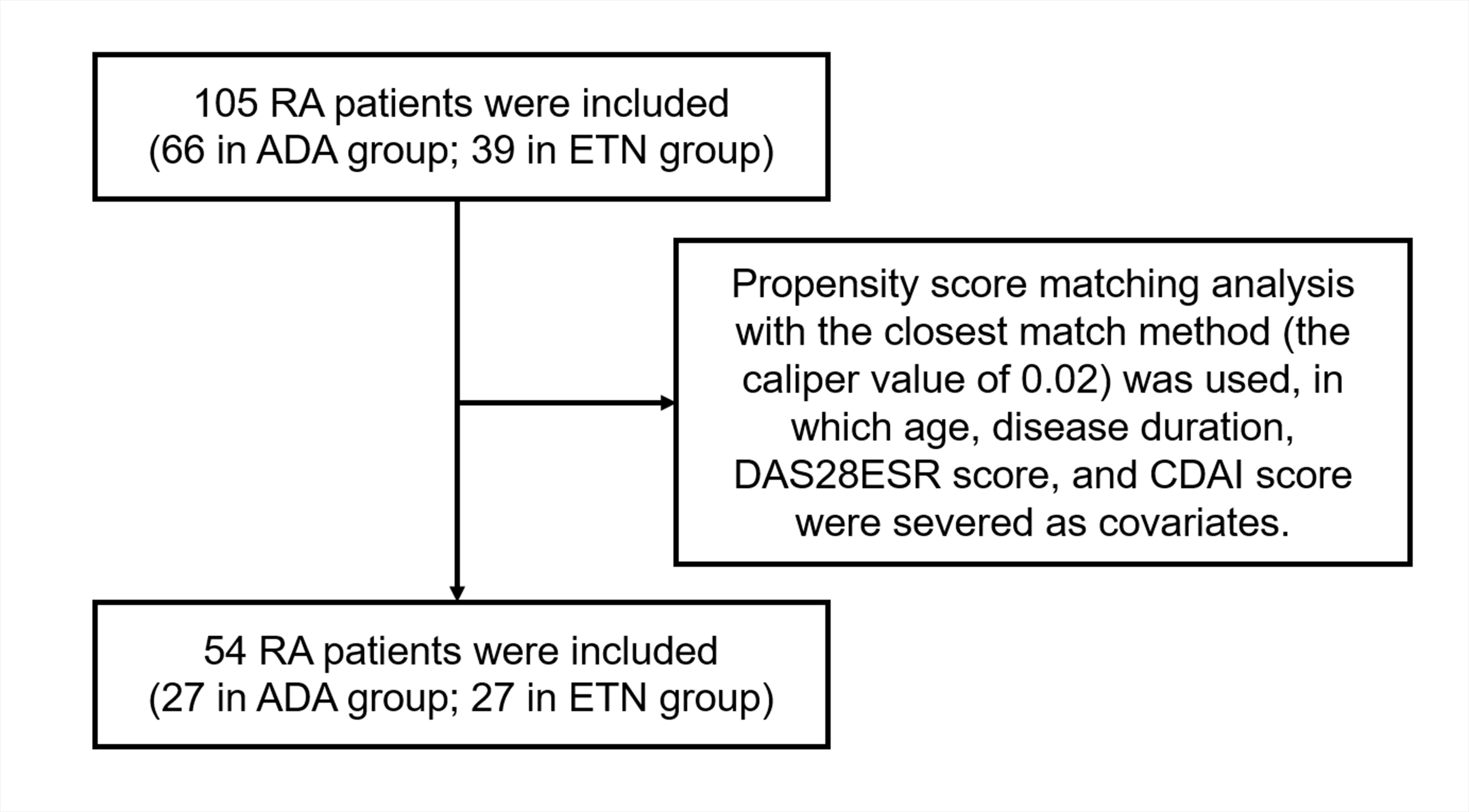

Supplement: Supplementary file 1 — Supporting Information [file IID3-12-e1166-s002.tif]

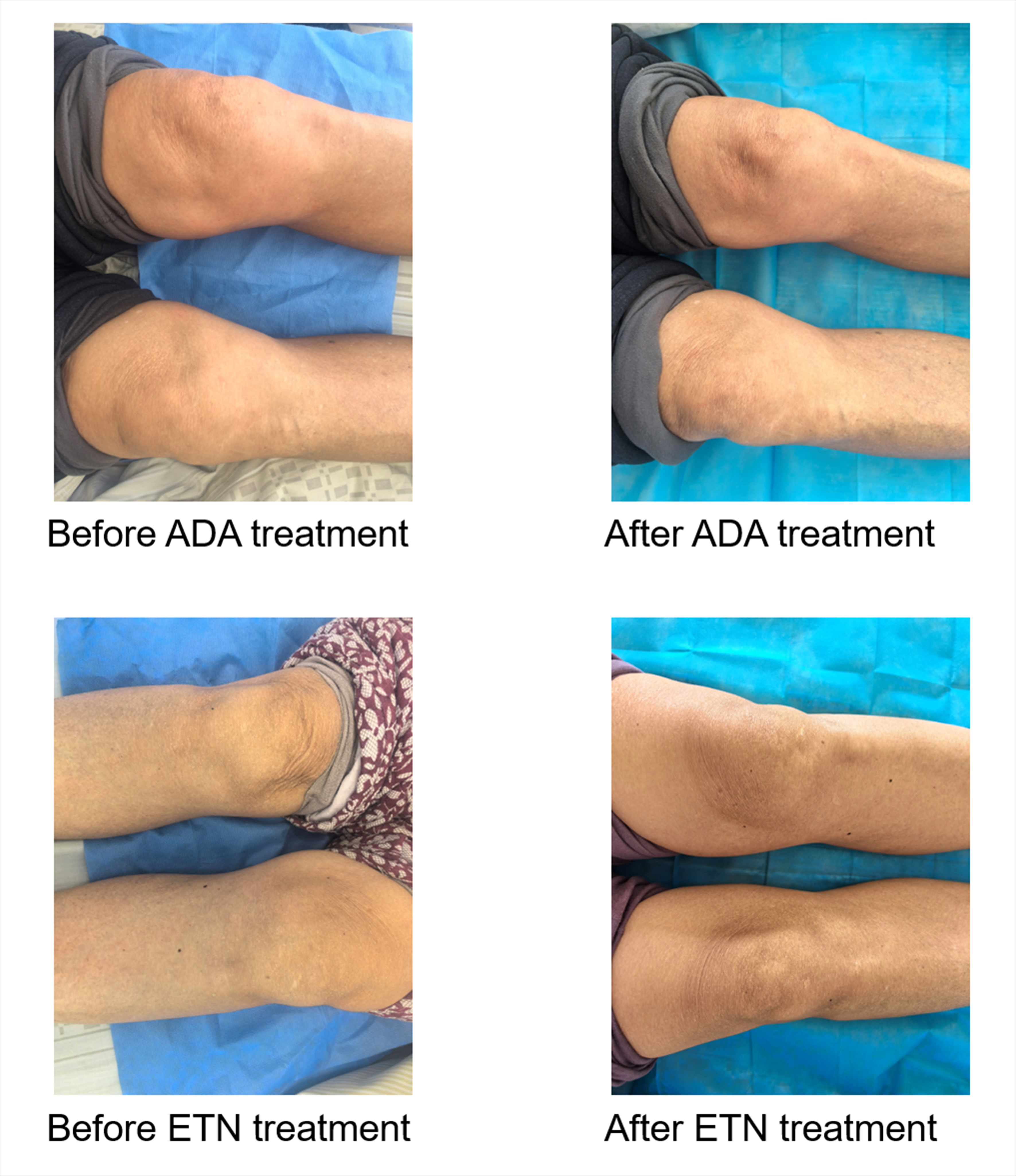

Supplement: Supplementary file 2 — Supporting Information [file IID3-12-e1166-s001.tif]
